# Challenges of heat stress on intestinal health in pig husbandry

**DOI:** 10.1016/j.fmre.2025.06.006

**Published:** 2025-06-16

**Authors:** Xiaolan Fan, Xue Tian, Mingzhou Li

**Affiliations:** a State Key Laboratory of Swine and Poultry Breeding Industry, Sichuan Agricultural University, Chengdu 611130, China; b Livestock and Poultry Multi-omics Key Laboratory of Ministry of Agriculture and Rural Affairs, College of Animal Science and Technology, Sichuan Agricultural University, Chengdu 611130, China

**Keywords:** Heat stress, Pigs, Intestinal injury, Intestinal barrier, Mitigation strategies

## Abstract

Global warming has intensified the threat of heat stress in pig husbandry, with the intestine emerging as a primary target of heat-induced injury. This perspective reviews recent advances in our understanding of the complex effects of heat stress on porcine intestinal health. We highlight current intervention strategies implemented in pig farming to enhance resilience against heat stress and mitigate associated intestinal damage. These strategies offer promising avenues to preserve pig health and improve productivity under rising environmental temperatures.

Heat stress has emerged as a major challenge for the livestock industry in the face of global warming. Pigs are particularly vulnerable to elevated temperatures due to their high metabolic rate and the absence of functional sweat glands. Exposure to heat stress significantly reduces production performance, as evidenced by decreased feed intake, slower weight gain, and elevated mortality rates. It also damages multiple organ systems, with the intestine being especially susceptible. Heat stress compromises the intestinal barrier by inducing extensive shedding of epithelial cells, which increases intestinal permeability and promotes bacterial translocation. In this perspective, we review recent advances in understanding how heat stress affects intestinal health in pigs. We focus on intervention strategies in pig husbandry aimed at enhancing the pig’s resilience to heat stress by preventing intestinal injury and promoting recovery.

## 1 Heat stress influences pig production and economic returns

**Fig. 1:**
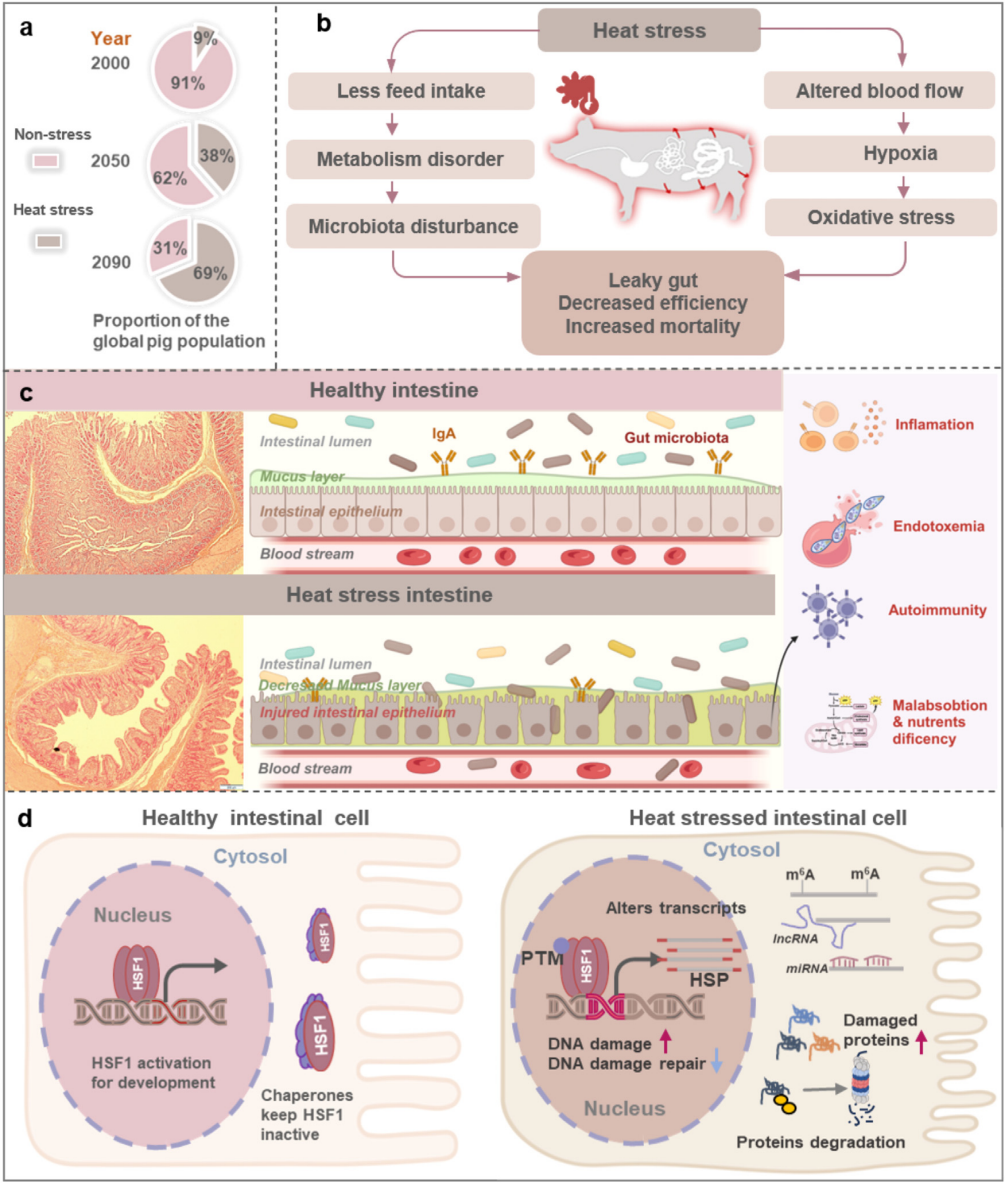
**Effects of heat stress on intestinal health during pig husbandry.** (a) Estimated proportion of the global pig population experiencing at least one day of extreme heat stress, which is defined by a temperature-humidity index threshold of 92, in 2000, 2050, and 2090 (Data from Thornton et al. [2]). (b) Heat stress-induced physiological changes in pig intestines. (c) Hematoxylin and eosin (HE) staining of pig jejunum sections (left), illustrating changes in intestinal villi and crypts after heat stress (Scale bar, 200μm). Schematic diagram depicting effects of heat stress on intestinal cells (middle). Compared to healthy pig intestines, those exposed to heat stress exhibit compromised intestinal barrier function, disruptions in intestinal microbiota, and translocation of endotoxins into the bloodstream. These changes may lead to systemic inflammation and altered immune responses (right). (d) Mechanisms by which intestinal cells respond to heat stress. HSP: heat shock protein, PTM: post-translational modifications.

Compared to pre-industrial levels (1850–1900), the global average temperature increased by 1.1 °C between 2011 and 2020 and is projected to surpass the 1.5 °C threshold by 2030 [1]. As a climate-sensitive sector, pig husbandry faces significant adverse effects from heat stress. In 2000, approximately 9% of pigs worldwide experienced at least one day of heat stress. Projections suggest this percentage will rise to 38% by 2050 and could reach 69% by 2090 (Fig. 1a) [2]. In the United States alone, annual economic losses from heat stress in pig farming are estimated at $300 million. These trends indicate that heat stress will continue to threaten the productivity and economic sustainability of global pig husbandry.

## 2 The intestinal tract: A target organ for heat stress injuries

The intestinal tract is highly susceptible to heat stress, which induces a range of pathological changes that compromise its structure and function. In response to elevated environmental temperatures, the body redirects blood flow away from internal organs toward the skin to facilitate heat dissipation. This redistribution reduces visceral blood flow by 30%–50%, leading to intestinal ischemia and hypoxia, which in turn weaken the intestinal barrier (Fig. 1b). Additionally, heat stress elevates circulating levels of stress hormones, particularly cortisol, and disrupts the tight junctions between intestinal epithelial cells. This disruption increases intestinal permeability, a condition commonly referred to as “leaky gut”.

Heat stress-induced “leaky gut” exerts widespread effects on the intestine, primarily impairing barrier integrity and disrupting nutrient absorption. Damage to the intestinal barrier includes multiple components: weakening of the mechanical barrier, thinning of the chemical layer, disturbance of the microbial community, and dysregulation of immune defenses (Fig. 1c). The mechanical barrier mainly consists of intestinal mucosal epithelial cells. Heat stress compromises this barrier by inducing epithelial cell death and disrupting tight junction (TJ) proteins. In pigs, heat stress reduces intestinal villus height by 20%–40% and deepens crypts, resulting in a significant decline in the villus-to-crypt (V/C) ratio (Fig. 1c, left panel). This structural damage severely compromises absorptive capacity. Moreover, epithelial degradation is recognized as a major contributor to heat stress-induced mortality. Extensive studies demonstrate that intestinal injury under heat stress involves a complex network of processes, including stress signaling, oxidative damage, TJ protein degradation, and excessive epithelial cell death. These interconnected pathways collectively drive intestinal dysfunction and compromise host health.

Intestinal mucus plays a critical role in the chemical barrier and is rich in components such as mucins. Goblet cells secrete mucins—glycoproteins primarily composed of mucopolysaccharides and proteins—which form a protective layer over the intestinal epithelium. Heat stress significantly downregulates mucin-related gene expression, resulting in thinning or complete loss of the mucus layer [3]. This disruption compromises intestinal barrier integrity and adversely affects the gut microbiota. Heat stress alters microbial composition, reduces microbial diversity, and triggers intestinal inflammation (Fig. 1c, middle panel). These changes particularly affect beneficial bacteria such as *Lactobacillus* and *Bifidobacterium*, whose populations decline markedly under heat stress. In contrast, opportunistic pathogens like *Escherichia coli* and *Salmonella* may proliferate due to the disrupted intestinal environment and weakened host immunity [4]. As the barrier deteriorates, endotoxins can translocate into the bloodstream, triggering Systemic Inflammatory Response Syndrome (SIRS). This systemic inflammation causes cellular injury in distant organs and represents a major contributor to heat stress-related mortality (Fig. 1c, right panel). Collectively, these findings highlight the gastrointestinal tract as a central driver of organ dysfunction and a key factor in heat stress-induced illness.

The intestinal villi are densely lined with nutrient transport proteins essential for efficient absorption. Heat stress damages the structural integrity of these villi, markedly reducing the absorptive surface area and directly impairing nutrient uptake. This loss of function disrupts systemic nutrient metabolism and contributes to broader physiological imbalances. In addition, heat stress suppresses the expression of genes involved in the synthesis of key digestive enzymes within intestinal epithelial cells. This includes a reduction in the production and secretion of trypsin, amylase, and lipase. As a result, both the enzymatic breakdown and absorption of nutrients are compromised. These changes underscore the intestine’s vulnerability as a primary target of heat stress, affecting both its structural morphology and functional capacity. Given the intestine’s central role in preserving homeostasis, such damage can propagate through inter-organ networks and contribute to widespread systemic dysfunction.

## 3 Mechanisms underlying heat stress-induced intestinal injury

Heat stress profoundly disrupts the entire flow of genetic information from DNA to protein (Fig. 1d). It intensifies oxidative stress by activating NADPH oxidase (NOX) and suppressing antioxidant enzymes such as superoxide dismutase (SOD) and glutathione peroxidase. This imbalance leads to DSBs and reduces the expression of key DNA repair proteins, including BRCA1 and Ku70, thereby impairing DNA repair efficiency. Three-dimensional chromatin analyses reveal that heat stress alters chromatin accessibility and disrupts the cell cycle in human K562 cells [5]. Furthermore, it induces genomic instability by promoting telomere shortening, inhibiting telomerase

Heat stress also affects transcriptional and post-transcriptional regulation. Transcription factor HSF1 activates the expression of heat-shock protein genes, a hallmark of the cellular response to thermal stress [4]. Additionally, the YAP/TAZ transcriptional co-activators, part of the Hippo signaling pathway, contribute to heat stress-induced transcriptional changes [6]. At the translational level, heat stress induces ribosomal pausing, selectively modulating the synthesis of splicing regulators such as SRSF1, and thereby influencing pre-mRNA splicing. Non-coding RNAs, including microRNAs (miRNAs) and long non-coding RNAs (lncRNAs), also play critical roles in the heat stress response. These molecules regulate transcription factors and bind to target genes, forming a complex regulatory network that coordinates gene expression under stress conditions.

Heat stress profoundly disrupts protein homeostasis by promoting the accumulation of misfolded or damaged proteins in the cytoplasm, thereby impairing cellular functions. HSPA1 plays a central role in enhancing cellular tolerance to heat stress. It recruits the 26S proteasome to the 80S ribosome, facilitating the degradation of aberrant peptide chains generated under stress and subsequently improving translational efficiency. Heat stress also induces targeted ubiquitination of specific proteins, a modification essential for cellular recovery. This process supports critical functions such as messenger ribonucleoprotein (mRNP) remodeling, stress granule disassembly, restoration of nuclear-cytoplasmic transport, and reactivation of protein synthesis [7]. Post-translational modifications further regulate the phase behavior of HSF1, a key transcription factor in the heat shock response. Phosphorylation of HSF1 at specific residues enhances its ability to undergo phase separation under stress conditions, thereby increasing its chromatin targeting capacity and transcriptional activation of heat shock protein (HSP) genes. Collectively, these findings underscore the extensive impact of heat stress on the full spectrum of gene expression—from DNA to protein—highlighting the potential for profound and lasting changes in cellular activity and fate in response to heat stress-induced damage.

## 4 Strategies for alleviating heat stress-induced intestinal injuries

Current strategies to mitigate heat stress in pig husbandry tend to be passive and broadly applied. Since the intestine plays a central role in heat stress-induced pathology, enhancing pigs’ resistance by minimizing or repairing intestinal injury in high-temperature environments offers a promising approach. Key strategies include deploying intelligent Internet of Things (IoT) systems for precise environmental and feeding control, strengthening the intestinal barrier, modulating immunity through the gut microbiota and its metabolites, and regulating body temperature via the gut-brain axis.

**Fig. 2:**
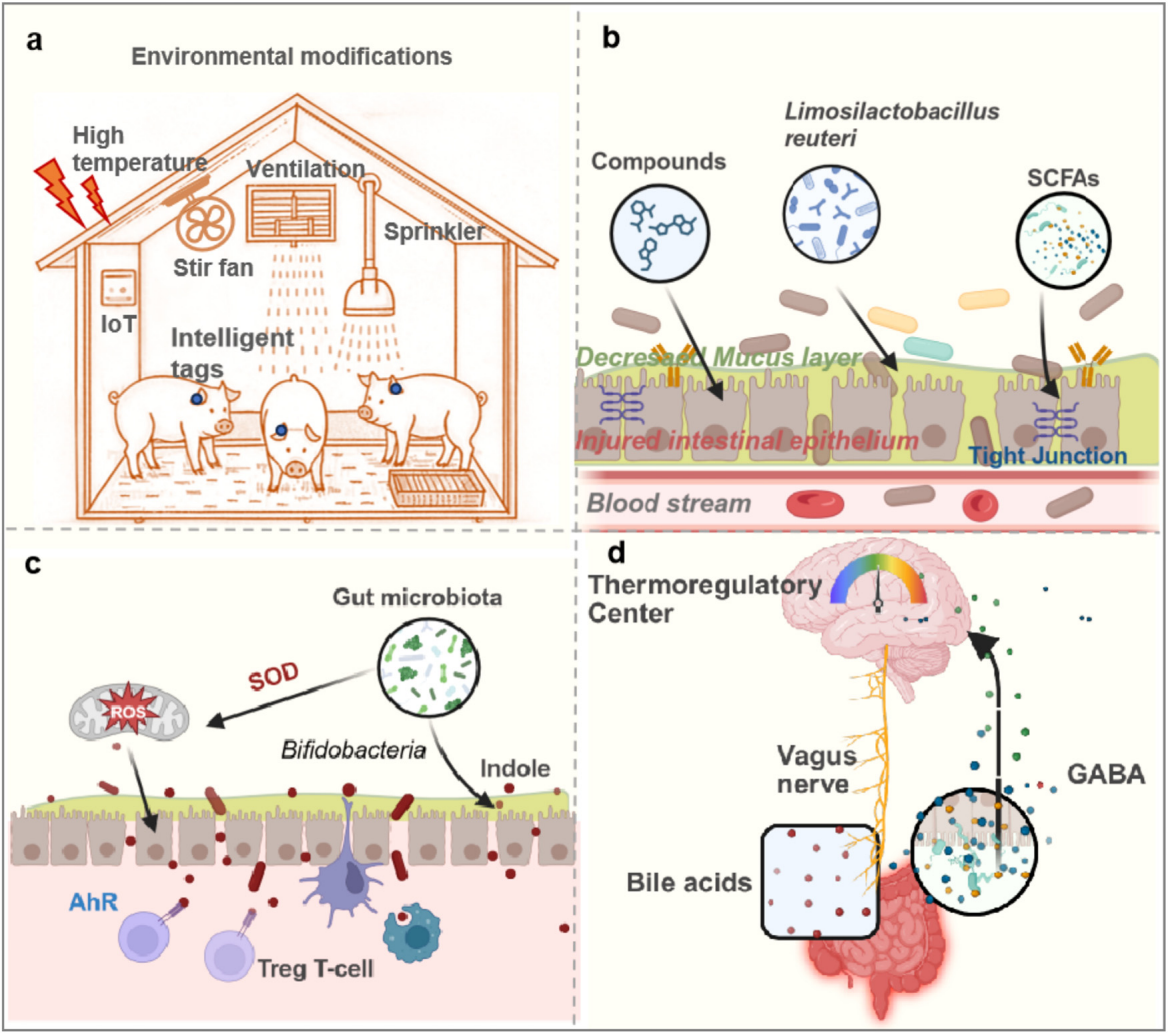
**Schematic diagram of strategies for alleviating heat stress-induced intestinal injuries.** (a) Improving the breeding environment through an intelligent IoT system that prevents intestinal damage in pigs caused by heat stress (Intelligent tags: Smart ear tags/collars, integrated with acceleration sensors and body temperature monitoring modules that track pig feed intake, water consumption, activity levels, and changes to body temperature in real time). (b) Mitigating or restoring heat stress-induced intestinal injuries by strengthening the intestinal barrier (Compounds: Quercetin, l-arginine et al., SCFAs: short-chain fatty acids). (c) Alleviating heat stress damage by reshaping the balance of gut microbiota or immune responses. (d) Regulating body temperature during heat stress using the gut-brain axis regulatory mechanism (GABA:γ-aminobutyric acid).

IoT technology can transform heat stress management in pig farming by shifting from passive responses to active prevention and control. Multi-dimensional data acquisition systems—incorporating temperature and humidity sensors as well as carbon dioxide and ammonia detectors—can monitor pig housing conditions in real time. Coupling these environmental metrics with physiological indicators such as breathing rate and body surface temperature, measured by infrared thermal imaging, allows the construction of an environmental-physiological association model. This model links to an intelligent control system that operates fans, wet curtains, and atomization cooling devices to dynamically regulate temperature and humidity within pig housing (Fig. 2a). According to Chinese climate models, investments in such technologies and improvements in rearing environments can reduce heat stress-related losses in the pig industry by approximately 21% [8]. In addition, lighting control systems can mimic natural circadian rhythms, mitigating heat stress impacts on pigs’ biological clocks. Intelligent ear tags or collars further enable real-time monitoring of feed and water intake, activity levels, and body temperature, facilitating early warning and timely identification of affected animals.

Heat stress disrupts the intestinal barrier by damaging the mucus layer and epithelial cells and downregulating TJ proteins. This compromise in barrier integrity impairs intestinal function. Strategies that protect the mucus layer and epithelial cells while upregulating TJ proteins can significantly enhance barrier function under heat stress conditions (Fig. 2b). Probiotics such as *Limosilactobacillus reuteri* promote mucin (MUC2) secretion and inhibit pathogen colonization. Quercetin, a plant-derived flavonoid, alleviates heat-stress-induced damage by activating the NRF2 pathway, which increases antioxidant enzyme activity by 50% [9]. Supplementing with butyrate, a short-chain fatty acid (SCFA), upregulates TJ protein expression through AMPK pathway activation. Nutritional interventions are widely recognized as effective countermeasures against heat stress-induced intestinal damage. For example, dietary supplementation with 1% l-arginine improves jejunal villus height and enhances the expression of occludin and porcine β-defensin 2 (PBD2) in finishing pigs exposed to intermittent heat stress [10]. These effects partially restore intestinal morphology and epithelial barrier function under heat stress.

Heat stress disrupts intestinal microbial balance and immune function in pigs. One promising approach to restore intestinal immune homeostasis involves using microbial preparations or their metabolites (Fig. 2c). High temperatures trigger an increase in mitochondrial reactive oxygen species (ROS) within intestinal cells. However, probiotics such as *Bifidobacteria* can enhance mitochondrial SOD activity and reduce oxidative damage. Additionally, the gut microbiota metabolizes tryptophan into indole compounds, which activate the aryl hydrocarbon receptor (AhR) and promote the differentiation of regulatory T cells (Treg) [11]. This pathway suppresses excessive immune responses and helps reestablish intestinal homeostasis following heat stress.

Heat stress also raises pigs' core body temperature significantly. Modulating the central thermoregulatory system via the gut–brain axis offers another potential strategy (Fig. 2d). Microbial metabolites, such as bile acids, relay signals to the central nervous system through the vagus nerve, thereby influencing the hypothalamic thermoregulatory center [12]. Moreover, *Lactobacillus rhamnosus* produces gamma-aminobutyric acid (GABA), which dampens sympathetic nervous system overactivity and decreases the release of thermogenic hormones like adrenaline. This gut-derived neurotransmitter regulation supports heat stress mitigation through central thermoregulation.

## 5 Current challenges and outlook

The structural and functional complexity of the intestine poses major challenges in using it as a target to mitigate heat stress in pigs. The effectiveness of probiotics, for example, depends on several factors, including the host’s baseline microbiota, the timing of administration, and the dosage. Administering probiotics during the early stages of heat stress may produce more favorable outcomes. However, when severe intestinal leakage occurs, immediate restoration of the intestinal barrier becomes a priority. Although some natural products have shown potential in alleviating heat stress by modulating host–microbiota interactions, their bioavailability and dose–response relationships remain insufficiently understood and require further investigation.

Improving intestinal health to enhance resistance to heat stress involves a complex network of biological processes, including microbial metabolism, immune modulation, neural signaling, and energy homeostasis. Future research should integrate metagenomics, metabolomics, and single-cell transcriptomics - particularly focused on intestinal immune cell responses - to uncover the dynamic regulatory networks of the pig intestine under heat stress. These approaches will offer deeper insights into how specific microbial strains or metabolites modulate heat stress signaling pathways. In parallel, advancing real-time monitoring technologies and biosensors capable of assessing intestinal barrier integrity and microbiota dynamics will provide powerful tools to evaluate the physiological impacts of heat stress. A more detailed investigation into the induction of HSPs and their roles in thermal adaptation may reveal novel targets for genetic selection and breeding programs aimed at enhancing heat tolerance. Gene editing technologies offer the potential to generate heat-resistant pig lines, and their integration with optimized breeding strategies and IoT-based data analytics can further improve resilience to heat stress. Moving forward, interventions targeting intestinal health should adopt a paradigm of precision and dynamic regulation. This strategy must account for individual variability and environmental factors to construct a comprehensive, multi-dimensional regulatory network for effective heat stress mitigation.

## CRediT authorship contribution statement

**Xiaolan Fan:** Writing – review & editing, Writing – original draft. **Xue Tian:** Resources. **Mingzhou Li:** Writing – review & editing, Project administration.
